# Complete Genome Sequences of *Campylobacter hyointestinalis* subsp. *hyointestinalis* Strain LMG 9260 and *C. hyointestinalis* subsp. *lawsonii* Strain LMG 15993

**DOI:** 10.1128/genomeA.00665-16

**Published:** 2016-07-14

**Authors:** William G. Miller, Emma Yee, Mary H. Chapman

**Affiliations:** Produce Safety and Microbiology Research Unit, Agricultural Research Service, U.S. Department of Agriculture, Albany, California, USA

## Abstract

*Campylobacter hyointestinalis* is isolated primarily from ruminants and swine, but is also occasionally isolated from humans. *C. hyointestinalis* is currently divided into two subspecies, *C. hyointestinalis* subsp. *hyointestinalis* and *C. hyointestinalis* subsp. *lawsonii*. This study describes the first closed whole-genome sequences of *C. hyointestinalis* subsp. *hyointestinalis* isolate LMG 9260 and *C. hyointestinalis* subsp. *lawsonii* isolate LMG 15993.

## GENOME ANNOUNCEMENT

*Campylobacter hyointestinalis* is a nonthermotolerant campylobacter that has been isolated from cattle (1–6), sheep (3), pigs (7), and reindeer (8). These organisms also occasionally cause disease in humans (9–12). *C. hyointestinalis* is divided currently into two subspecies (13), *C. hyointestinalis* subsp. *hyointestinalis* and *C. hyointestinalis* subsp. *lawsonii*. Although most *C. hyointestinalis* strains are not identified to the subspecies level, we have almost exclusively isolated *C. hyointestinalis* subsp. *hyointestinalis* from cattle and *C. hyointestinalis* subsp. *lawsonii* from swine (data not shown). This study presents the first complete *C. hyointestinalis* genome sequences, for the human *C. hyointestinalis* subsp. *hyointestinalis* strain LMG 9260 and the porcine *C. hyointestinalis* subsp. *lawsonii* strain LMG 15993*.*

Roche GS-FLX and the Illumina HiSeq were used to complete both genomes. Roche 454 shotgun and paired-end reads were assembled, using the Roche Newbler assembler (version 2.6), into single scaffolds of 19 (*C. hyointestinalis* subsp. *hyointestinalis*) and six (*C. hyointestinalis* subsp. *lawsonii*) contigs. Sanger sequencing of contig-bridging amplicons was used to close each scaffold into a single contig. All 454 base calls were validated using Illumina HiSeq reads (SeqWright, Houston, TX). The final coverage for both strains was >700×. Optical restriction maps (OpGen, Gaithersburg, MD) with the restriction enzyme PvuII were used to validate both assemblies. Illumina HiSeq reads were also used to characterize hypervariable GC tracts, as described previously (14). Average nucleotide identity (ANI) analysis was performed using JSpecies version 1.2.1 (15).

The *C. hyointestinalis* strains have circular genomes of 1,753 kb with a similar G+C content (approximately 34%). No plasmids were identified. Protein-, rRNA- and tRNA-coding genes were identified as described previously (14), but using a BLASTP identity of 40% to define a positive match. The two genomes contain a similar number of putative protein-coding genes (1,678 for *C. hyointestinalis* subsp. *hyointestinalis* and 1,711 for *C. hyointestinalis* subsp. *lawsonii*) and pseudogenes (55 for *C. hyointestinalis* subsp. *lawsonii* and 59 for *C. hyointestinalis* subsp. *hyointestinalis*). The *C. hyointestinalis* genomes also contain a large number of hypervariable homopolymeric GC tracts (42 for *C. hyointestinalis* subsp. *lawsonii* and 52 for *C. hyointestinalis* subsp. *hyointestinalis*); however, although many are in genes associated with the biosynthesis of surface structures, restriction-modification (R/M) systems, and signal transduction, the majority in each genome are intergenic or located in genes encoding proteins of undetermined function.

The ANI between the two strains is 94.3%. This is below the proposed ANI cutoff value of 95% for species delineation (16). However, the *Campylobacter lari* and *Campylobacter fetus* subspecies (i.e., *C. fetus* subsp. *fetus/C. fetus* subsp. *venerealis*, and *C. fetus* subsp. *testudinum*) have lower intraspecies ANI values of 92 to 93% (data not shown), despite DNA-DNA hybridization (DDH) values of >70% (17, 18), suggesting that the correspondence between ANI and DDH in *Campylobacter* should be readdressed.

The *C. hyointestinalis* subsp. *hyointestinalis* and *C. hyointestinalis* subsp. *lawsonii* genomes are moderately syntenic, with similar gene contents. The gene set common to both strains is approximately 84% of the genes in each genome. The variable gene set includes genes that encode surface structure biosynthesis proteins and R/M systems, genes in integrated elements (such as a putative Mu-like phage in *C. hyointestinalis* subsp. *lawsonii*), and clustered regularly interspaced short palindromic repeat (CRISPR) arrays. Insertion sequences (IS) are present in both strains, with *C. hyointestinalis* subsp. *hyointestinalis* containing five IS elements of either the IS*605*/IS*607* or IS*1595* family.

### Nucleotide sequence accession numbers.

The complete *C. hyointestinalis* genome sequences of *C. hyointestinalis* subsp. *hyointestinalis* strain LMG 9260 and *C. hyointestinalis* subsp. *lawsonii* strain LMG 15993 have been deposited in GenBank under the accession numbers CP015575 and CP015576, respectively.
